# Correlation of clinical and biological parameters in endogenous psychoses developed after coronavirus infection (COVID-19)

**DOI:** 10.1192/j.eurpsy.2023.1258

**Published:** 2023-07-19

**Authors:** S. A. Zozulya, S. V. Sizov, I. V. Oleichik, T. P. Klyushnik

**Affiliations:** 1 Laboratory of Neuroimmunology; 2 Department of Endogenous Mental Disorders and Affective States, Mental Health Research Centre, Moscow, Russian Federation

## Abstract

**Introduction:**

The importance of inflammation as a common pathophysiological mechanism underlying infectious and non-infectious diseases determines the relevance of studying the effect of coronavirus infection (COVID-19) on the course of endogenous psychoses.

**Objectives:**

Clinical and immunological study of the potential impact of coronavirus infection on the course of endogenous psychoses.

**Methods:**

Two groups of female patients aged 16 to 48 years with depressive-delusional conditions (F20.01, F21, F31) developed after a coronavirus infection were examined. In 15 patients, psychosis developed 1-2 months after COVID-19, and in 18 patients it occured within 2-6 months after the disease. The severity of psychopathological symptoms was assessed using PANSS and HDRS-21 scales. The activity of inflammatory markers leukocyte elastase (LE) and α1-proteinase inhibitor (α1-PI) in the blood of patients was determined. Neutrophil count, lymphocyte count and neutrophil/lymphocyte ratio were calculated. The standard values of indicators of healthy donors corresponding to patients by age and sex were used as controls.

**Results:**

Endogenous psychoses that developed later after coronavirus infection were associated with “typical” inflammation, increased α1-PI activity and increased neutrophil degranulation (by LE activity) compared to normal values. Patients with this immune profile were observed to develop depressive-delusional states with a prevalence of positive affectivity (anxiety, melancholy) and an expanded nature of delusional disorders that were predominantly non-congruent with affect. Endogenous psychoses that developed within 2-6 months after COVID-19 were characterized by decreased neutrophil count, decreased LE activity, prevalence of negative affectivity (apathy, asthenia, adynamia), and a relatively undeveloped nature of delusional disorders that were predominantly congruent with affect.

**Conclusions:**

Clinical and biological correlates presumably indicate the modulating effect of the coronavirus infection on (neuro)inflammation and the structure of endogenous psychoses.

**Disclosure of Interest:**

None Declared

